# A feasibility study exploring the role of pre-operative assessment when examining the mechanism of ‘chemo-brain’ in breast cancer patients

**DOI:** 10.1186/s40064-016-2030-y

**Published:** 2016-03-31

**Authors:** Valerie Jenkins, Ryan Thwaites, Mara Cercignani, Sandra Sacre, Neil Harrison, Hefina Whiteley-Jones, Lisa Mullen, Giselle Chamberlain, Kevin Davies, Charles Zammit, Lucy Matthews, Helena Harder

**Affiliations:** Sussex Health Outcomes Research and Education in Cancer (SHORE-C), Brighton and Sussex Medical School, University of Sussex, Brighton, UK; Brighton and Sussex Medical School, University of Sussex, Brighton, UK; Clinical Imaging Sciences Centre (CISC), Brighton and Sussex Medical School, University of Sussex, Brighton, UK; Brighton and Sussex University Hospitals NHS Trust, Brighton, UK

**Keywords:** Breast cancer, Cytokines, Brain imaging, Cognitive function, Quality of life, Fatigue

## Abstract

**Background:**

Women receiving chemotherapy treatment for breast cancer may experience problems with their memory and attention (cognition), which is distressing and interferes with quality of life. It is unclear what causes or contributes to the problems they report: psychological distress, fatigue, coping style, or specific biological changes for example to pro inflammatory cytokines. Research shows however, that approximately a third of women with breast cancer perform poorly on tests of cognition before commencing chemotherapy. We aimed to examine the acceptability and relevance of pre-surgical assessments (bloods, brain imaging, cognitive tests and self-report questionnaires) when investigating the phenomenon of ‘chemo-brain’ and investigate whether inflammatory markers mediate chemotherapy-induced neuropsychological impairments in women treated for breast cancer.

**Methods:**

Women with early stage breast cancer completed neuropsychological and quality of life assessments at T1 (pre-surgery), T2 (post-surgery before chemotherapy) and T3 (6 months later). Blood cytokine levels were measured at the same time points and brain imaging was performed at T1 and T3.

**Results:**

In total, 14/58 women participated (8 chemotherapy, 6 non-chemotherapy). Prior to the start of chemotherapy a decline in cognitive performance compared to baseline was observed in one participant. At T3 women who received chemotherapy reported poorer quality of life and greater fatigue. Increases in soluble tumour necrosis factor receptor II (sTNFRII), interleukin-6, interleukin-10 and vascular endothelial growth factor occurred post chemotherapy only. Levels of sTNFRII were inversely correlated with grey matter volume (GMV) of the right posterior insula in both groups. At T3, the chemotherapy group displayed a greater reduction in GMV in the subgenual and dorsal anterior cingulate, and the inferior temporal gyrus.

**Conclusions:**

Pre-operative recruitment to the study was challenging; however, the lack of significant changes in blood cytokine levels and neuropsychological tests at T2 implies that post surgery may be a valid baseline assessment, but this needs further investigation in a larger study. The preliminary results support the hypothesis that chemotherapy induced fatigue is mediated by a change in peripheral cytokine levels which could explain some symptoms of ‘chemo brain’ experienced by patients.

## Background

‘Chemo-brain’ is a term used for cognitive impairment such as memory and attention problems, following chemotherapy. Previous neurological studies show cognitive dysfunction in 15–70 % of patients treated with chemotherapy (Ahles et al. 2002; Jim et al. 2009; Hermelink et al. 2007; Jenkins et al. 2006; Wefel and Schagen 2012). The wide variance is partly due to the use of different neuropsychological tests between studies, different reference data and performance cut offs for classifying test results (Shilling et al. 2006). According to the International Cognition and Cancer Task Force (ICCTF), studies conducted to date show a frontal subcortical profile with the following domains affected: learning and memory, processing speed and executive function (Wefel et al. 2011). While chemotherapy may be a key contributor, it is not the sole cause of cognitive impairment as a third of women perform poorly on cognitive tasks following surgery but prior to starting adjuvant therapies (Hermelink et al. 2007; Ahles et al. 2008). Poor performance has been associated with cancer related stress similar to that observed in post-traumatic stress disorder (Reid-Arndt and Cox 2012; Hermelink et al. 2015). Whatever the underpinning causal factors, the phenomenon of ‘chemo brain’ continues to cause problems for patients and impact negatively on their quality of life (QoL).

The mechanisms underlying chemo-brain are not clear but suggestions include stress and coping styles (Reid-Arndt and Cox 2012), direct neurotoxic injury, telomere shortening, oxidative stress, cytokine dysregulation, oestrogen-mediated effects, and genetic polymorphisms (Walker et al. 2012). Chemotherapy drugs, which because of their molecular size are mostly unable to cross the blood–brain-barrier can cause toxicity to the brain indirectly via cytokines that do cross over. Evidence suggests that elevated levels of peripheral cytokines may be related to cognitive problems in cancer patients (Ahles and Saykin 2007; Janelsins et al. 2012; Wang et al. 2015). Studies employing magnetic resonance imaging (MRI) and functional MRI (fMRI) techniques demonstrate both hypo- and hyper-activation in the absence or presence of cognitive dysfunction. Imaging studies have documented structural changes to the brain, including reduction in grey matter volume (GMV) and white matter microstructural damage (Wefel and Schagen 2012; McDonald et al. 2012). Alterations are noted from 1 month to 21 years post chemotherapy (McDonald et al. 2012; Koppelmans et al. 2011). However, some structural differences may already be present before starting treatment (Scherling et al. 2012) and more recently symptoms of fatigue have been associated with the observed abnormalities (Menning et al. 2015).

One of the criticisms of research investigating chemo-brain is the lack of a preoperative baseline assessment in order to be confident that any changes found post treatment are not confounded by surgery (e.g., type, length of general anaesthesia, stress). This is especially important when investigating changes to levels in cytokines and brain imaging. To the best of our knowledge, this is the first study in breast cancer patients to combine neuroimaging, blood tests, cognitive testing and questionnaires reporting cognitive symptoms, fatigue, QoL, and anxiety/depression before and after surgery, and following a course of chemotherapy.

The primary objective was to explore the acceptability and feasibility of conducting a comprehensive assessment (neuropsychological assessment, blood tests and MRI scan) prior to breast cancer surgery. Secondary objectives were: (1) to examine whether changes occur in cytokine levels following surgery and chemotherapy compared to baseline (pre-surgery) and whether these correlate with changes in subjective cognitive performance, fatigue and imaging data, and (2) to investigate using advanced imaging techniques if chemotherapy is associated with quantifiable structural brain changes and if this is related to changes in cognitive function and/or magnitude of inflammatory response to chemotherapy.

## Methods

### Study design and participants

This was a prospective, longitudinal study involving objective and subjective measures of cognitive function and QoL, together with blood cytokine analysis prior to surgery (T1), following surgery and before adjuvant treatment (T2) and approximately 6 months later at completion of chemotherapy treatment (T3). Brain imaging with highly sensitive MRI techniques was conducted at T1 and T3 only.

Participants were women aged 47–61 years diagnosed with early stage invasive breast cancer (who may or may not require chemotherapy treatment) and those with ductal carcinoma in situ. Patient identification and eligibility screening took place in the breast cancer Multidisciplinary Team Meetings and clinics of the Brighton and Sussex University Hospitals NHS Trust. Those eligible were given an information pack and letter of introduction following their surgical consultation and contacted the research team if they wanted to participate. The study was approved by the London Camberwell St Giles National Regional Ethics Committee (13/LO/0164) and written consent was obtained from all participants.

### Procedures and measures

Participant characteristics and treatment-related data were collected at baseline by interview and via medical records. Premorbid intelligence was estimated with the National Adult Reading Test (Nelson and Wilson 1991; Crawford et al. 1989). At T3 women were asked to provide feedback about study participation, specifically the timing of the assessments, which were conducted at the University of Sussex and took approximately 3 h to complete.

### Neuropsychological assessments

A comprehensive test battery was selected according to the recommendations of the International Cognition in Cancer Task Force (ICCTF) to assess cognitive function (Wefel et al. 2011) using the following 6 measures: Rey Auditory Verbal Learning Test (RAVLT) (Schmidt 1996), Letter–Number Sequencing (LNS) (Wechsler 1997), Stroop Colour and Word Test (Golden 1978); Controlled Oral Word Association test (COWAT) (Benton et al. 1994; Shao et al. 2014), Double Letter Cancellation Task (DLCT) (Lezak 2012), and the Grooved Pegboard (GP) (Lafayette 1998). Tests were administered in the same order at each time point and alternate versions used where possible to minimise practice effects (“Appendix 1”).

### Patient reported outcomes (PROs)

Quality of life was measured with the Functional Assessment of Cancer Therapy-Breast (FACT-B) version 4 (Brady et al. 1997). This is a 37-item self-administered questionnaire with 4 subscales: physical well-being (PWB), social well-being (SWB), emotional well-being (EWB), functional well-being (FWB), and a breast cancer-specific subscale (BCS). Each item has a 5-point response scale ranging from 0 (not at all) to 4 (very much). Higher total scores indicate better functioning. The 13-item Fatigue subscale version 4 was used to measure perceived fatigue over the last week (Yellen et al. 1997). Perceived cognitive function was assessed with the FACT-Cognitive function (FACT-Cog) version 3 (Wagner et al. 2009). This scale has 37 items for 4 subscales: perceived cognitive impairments (PCI), perceived cognitive abilities (PCA), deficits observed or commented on by others (Oth), and impact on QoL (CogQOL). The 10-item Trauma Screening Questionnaire (TSQ) was included to screen for symptoms of traumatic stress (Brewin et al. 2002).

### Blood collection and biological specimens

Serum was collected from clotted peripheral blood collected at each time point by venepuncture into silica containing vacutainers (Becton–Dickinson, Oxford, UK). Serum levels of Interleukin-6 (IL-6), IL-10, monocyte chemotactic protein-1 (MCP-1), brain-derived neurotrophic factor (BDNF) and soluble TNF receptor II (sTNFRII) were measured by Quantikine high sensitivity ELISAs (R&D systems, Minneapolis, USA). Vascular endothelial growth factor (VEGF) was measured using Duoset antibodies (R&D systems, Minneapolis, USA) according to manufacturer’s instructions. Haemoglobin levels were recorded separately as part of a participant’s clinical assessment by the clinical team.

### MRI acquisition

All imaging was performed using a research optimised 1.5T scanner (Siemens Magnetom Avanto) with a 32-channel head coil. Each session lasted approximately 1 h and included the following scans: (1) a T1-weighted high-resolution (MPRAGE) volumetric scan, for measuring white and grey matter regional volume changes; (2) a diffusion-tensor imaging (DTI) sequence (60 directions, maximum b factor = 1200 s/mm^2^); (3) a serial gradient echo echo-planar imaging (EPI) scan to record resting-state (RS) fMRI data (approximately 7 min); (4) a 3D gradient echo sequence, which collects a series of magnetization transfer (MT)-weighted volumes with differing degrees of MT-weighting; (5) a T1-mapping sequence, and (6) a B1-mapping sequence. During the fMRI scan, subjects were instructed to lie still, with their eyes closed, without falling asleep. Only the results from the volumetric assessment are reported here.

### Statistical methods

Demographical and clinical characteristics for all patients are summarised and stratified by chemotherapy exposure status. The feasibility study was not powered for comparison between groups on the objective cognitive tasks; results are presented as changes across time by determining the proportion of women with ≥2 test scores at or below 1.5 SDs, or ≥1 test score at or below 2.0 SDs from the normative mean, or both (Wefel et al. 2011). Also the reliable change index (RCI) with a correction for observed practice effects was calculated for each measure (Sawrie et al. 1996). The RCI is a well-known method used to determine if a change is reliable rather than simply due to test measurement error (i.e. practice effects), accounting for differences between pre- and post-test variance. An RCI criterion of ≥1.96 (95 % confidence interval) was utilized. An RCI was calculated for a selection of cognitive measures for each participant using the baseline and T2 and baseline and T3 data of an age and intelligence matched comparative group of control subjects and categorized as ‘Declined’, ‘Improved’, or ‘Unchanged’ (Jacobson and Truax 1991).

Changes in patient reported outcomes for the FACT-B and the Fatigue subscale were calculated. The trial outcome index (TOI) used for the FACT-B is an efficient summary index of physical/functional outcomes, and a common endpoint used in clinical trials (Eton et al. 2004). It is the sum of the scores of the 28 items included in the PWB, FWB and BCS (range 0–112). A change of at least seven points from baseline TOI score for FACT-B is considered a relevant clinically minimal important difference, and for the Fatigue subscale it is a change of ≥3 points (Yost and Eton 2005). A total TSQ score of ≥6 was considered to be an indication of the presence of traumatic stress (Brewin et al. 2002).

To assess the relationship between cytokine levels and cognitive impairment during chemotherapy, serum levels of sTNFRII, IL-6, VEGF, IL-10, BDNF and MCP-1 were measured. Significance was assessed using a one-tailed Mann–Whitney test using GraphPad Prism version 5 (GraphPad Software, La Jolla California USA).

The MPRAGE scans from all patients at T1 and T3 were analysed using voxel-based morphometry (VBM) (Ashburner and Friston 2000; Ashburner and Friston 2005). VBM is an image analysis technique which is used to identify regional difference in GMV between groups or to investigate the correlation between local GMV and other variables of interest (“Appendix 2”). The technique relies on the accurate alignment of images from different individuals into a common brain space, in order to achieve a perfect correspondence between a specific set of image coordinates and the same anatomical brain structure across subjects. The images are then segmented into separate tissues to provide “maps” of grey matter, white matter and volume. Statistical comparisons can then be carried out to highlight the areas of the brain where significant results are found. For the purpose of this paper, data were analysed using the VBM8 toolbox (http://dbm.neuro.uni-jena.de/vbm/) for SPM8 (Wellcome Trust Centre for Neuroimaging, http://www.fil.ion.ucl.ac.uk/spm/software/spm8/). The images were therefore segmented and normalised to Montreal Neurological Institute (MNI) space and segmented, then they were spatially filtered with a 3D Gaussian kernel (full width at half maximum = 8 mm^3^). The purpose of this final step, also known as ‘smoothing’, is to compensate for residual inter-individual differences and to make the data more normally distributed in preparation for statistical analysis. To explore changes in GMV induced by chemotherapy, a two-way between subject ANOVA model in SPM8 was used, within the framework of the general linear model. In a within-subject design, we modelled the effect of group (chemotherapy vs no chemotherapy) and the effects of session (T1 vs. T3). We also modelled the interaction between these two factors. A linear regression model was used to investigate the presence of any association between GMV at T3 and inflammatory factors. As this is an exploratory study, we accepted as significant p values inferior to 0.005, in clusters of 100 or more voxels.

## Results

### Patient characteristics

The research nurse approached 58 potentially eligible women for the study in clinic between May 2013 and April 2014; 9 were ineligible and 14/49 (29 %) participated. There was no study attrition at follow-up. The main reason for nonparticipation was lack of time; many women worked and were reluctant or unable to fit in the lengthy test procedures before surgery.

Table 1 displays participants’ characteristics. Median time between T1 and surgery was 8 days (range 2–22). Eight women were treated with chemotherapy (CT) and all received an anthracycline containing regimen. Pre-operative levels of haemoglobin were in the normal range (≥120 g/L) for all participants; 4/8 CT women were anaemic at T3. Four women were postmenopausal at baseline and seven experienced a treatment-induced menopause. Women in the no-chemotherapy group (NCT) had higher premorbid IQ (p = 0.015). At T3 86 % of women had received or were scheduled for radiotherapy and 71 % were prescribed endocrine therapy.

**Table 1:** Demographics and clinical characteristics

	Chemotherapy *n* = *8*	No chemotherapy *n* = *6*
Age (years)		
Mean (SD)	52.6 (3.9)	50.2 (2.3)
Range	50–61	47–53
Partner		
Yes	4	5
Education		
Higher	3	4
Further	2	1
Secondary	3	1
FSIQ^a^		
Mean (SD)*	111.1 (7.4)	120.8 (4.5)
Range	106–123	112–124
Employed		
Full-time	5	4
Part-time	3	2
Cancer stage*^,b^		
I	0	4
II	1	1
III	7	0
Type of surgery		
Mastectomy	2	1
WLE	6	5
Node sampling		
Yes	8	6
Chemotherapy regimen		
AC	1	–
FEC 75	2	–
FEC-T^c^	5	–
Herceptin		
Yes	2	–
Radiotherapy		
Yes	7	5
Endocrine therapy by T3		
Yes	3	6
Postmenopausal at baseline		
Yes	3	2
Prior HRT		
Yes	2	1
Baseline Hb level (g/L)		
Mean (SD)	135.4 (10.9)	133.8 (7.6)
Range	120–151	126–145

*AC* doxorubicin and cyclophosphamide, *BMI* body mass index, *FEC 75* fluorouracil, epirubicin (75 mg/m^2^) and cyclophosphamide, *FEC-T* fluorouracil, epirubicin, cyclophosphamide and docetaxel, *FSIQ* full scale intelligence quotient, *G-CSF* granulocyte-colony stimulating factor, *Hb* haemogloblin, *HRT* hormone replacement therapy, *SD* standard deviation, *WLE* wide local excision

* *p* < 0.05

^a^Derived from the National Adult Reading Test

^b^One participant had an ungraded tumour due to unusual presentation

^c^One woman had neo-adjuvant FEC-T chemotherapy

### Cognitive and PRO results

Outcomes of the neuropsychological tests and PROs by treatment group for each time point are listed in Table 2. A difference in performance compared to normative values on the objective tests were observed at T3 (after treatment completion) in two participants (one in each treatment group). The proportion of each group showing reliable decline or reliable improvement on the RAVLT, Stroop and LNS was calculated for T2 and T3. Reliable decline was observed in 1 CT patient; however this was prior to the start of chemotherapy (T2). No reliable improvement was observed for any participant.

**Table 2:** Cognitive performance (raw test scores) at each time point for CT and NCT groups: mean (SD)

	Chemotherapy (*n* = *8*)			No chemotherapy (*n* = *6*)		
	T1	T2	T3	T1	T2	T3
RAVLT						
Supraspan (0–15)	7.3 (2.3)	7.3 (2.7)	7.2 (2.2)	10.3 (3.3)	9.0 (3.0)	9.4 (3.0)
Total score (0–75)	56.0 (9.1)	52.4 (12.3)	54.2 (9.8)	62.7 (7.9)	61.3 (9.8)	61.8 (8.6)
Delayed score (0–15)	12.5 (2.8)	11.3 (2.4)	11.3 (2.4)	11.5 (1.5)	12.7 (2.4)	12.3 (2.0)
Double letter cancellation						
Time (s)	185.0 (32.1)	174.3 (20.1)	179.4 (69.5)	176.7 (26.8)	163.7 (30.3)	160.0 (30.4)
Total score	69.4 (3.3)	70.9 (2.1)	69.5 (2.9)	72.0 (3.3)	72.8 (2.6)	72.3 (3.2)
Letter number sequencing						
Total score	10.3 (3.2)	10.6 (3.7)	10.8 (3.3)	12.8 (3.0)	12.8 (2.6)	13.2 (3.0)
COWAT						
Total score	56.4 (15.5)	54.0 (16.6)	54.4 (15.0)	55.2 (9.9)	54.0 (6.5)	55.2 (9.0)
Stroop Colour Word Test						
Word card	101.1 (15.4)	101.0 (18.0)	97.8 (20.0)	113.2 (14.3)	114.7 (16.5)	113.8 (17.3)
Colour card	78.0 (11.7)	77.0 (15.1)	77.0 (11.3)	87.3 (12.8)	86.0 (9.1)	87.3 (14.7)
Colour-word card	45.3 (6.6)	47.1 (7.6)	47.5 (6.9)	52.0 (14.8)	55.8 (9.7)	56.3 (9.9)
Interference score	1.5 (4.8)	3.8 (7.2)	4.7 (8.1)	2.9 (12.0)	6.9 (8.6)	7.1 (8.1)
Grooved Pegboard						
Dominant hand: time (s)	67.1 (7.2)	65.7 (9.3)	66.2 (7.5)	63.3 (6.6)	59.0 (5.7)	61.2 (6.8)
Dominant hand: total	92.4 (7.4)	90.9 (9.3)	91.4 (7.7)	88.3 (6.6)	84.2 (5.7)	86.3 (6.8)
Non-dominant hand: time (s)	76.4 (8.7)	75.4 (7.6)	75.4 (7.4)	78.0 (10.5)	66.8 (4.2)	70.4 (9.5)
Non-dominant hand: total	101.4 (8.7)	100.4 (7.6)	100.5 (7.5)	103.5 (9.8)	91.8 (4.2)	95.6 (9.4)
FACT-B (0–112)	105.6 (7.8)	101.8 (17.5)	101.6 (12.9)	115.4 (10.8)	112.3 (13.3)	116.0 (18.0)
FACT-F (0–160)	114.6 (15.0)	111.8 (18.8)	107.5 (19.7)	128.9 (12.0)	125.6 (14.8)	127.2 (20.3)
FACT-Cog						
PCI (0–72)	49.9 (14.4)	42.9 (15.8)	36.8 (14.0)	49.8 (8.4)	47.2 (15.7)	48.0 (11.9)
PCA (0–28)	18.6 (4.3)	13.3 (4.8)	13.3 (5.6)	16.8 (6.2)	18.2 (5.7)	18.0 (6.4)
Oth (0–16)	14.3 (2.9)	13.9 (2.9)	14.9 (1.9)	14.8 (2.4)	14.8 (1.5)	15.2 (1.2)
CogQOL (0–16)	12.9 (3.8)	11.1 (2.9)	11.3 (3.8)	9.5 (5.4)	12.7 (1.8)	12.5 (4.0)
TSQ (0–10)	4.4 (2.5)	3.9 (2.2)	3.3 (1.3)	4.8 (2.5)	3.0 (2.8)	2.2 (1.9)

*RAVLT* Rey Auditory Verbal Learning Test, *COWAT* Controlled Word Association Test, *FACT-B* Functional Assessment of Cancer Therapy-Breast, *FACT-F* Functional Assessment of Cancer Therapy-Fatigue, *FACT-Cog* Functional Assessment of Cancer Therapy-Cognitive function, *PCA* perceived cognitive abilities, *PCI* perceived cognitive impairment, *Oth* deficits observed or commented on by others, *CogQOL* impact on quality of life, *SD* standard deviation, *TSQ* Trauma Screenings Questionnaire

During the study six patients [5 CT (63 %); 1 NCT (17 %)] experienced a decrease at T3 from baseline of ≥7 points in TOI of the FACT-B (i.e. reporting lower health-related QoL). Similar findings occurred for fatigue; 6 (75 %) CT and 2 (33 %) NCT. Levels of fatigue correlated with the subscale PCI from the FACT Cog (r = 0.848; p < 0.001). Improvement in health-related QoL was reported by four women (two in each group), and a significant reduction in fatigue for three participants (2 CT; 1 NCT). High levels of stress were reported by five women (3 CT, 2 NCT) at baseline, and two women (one in each group) post-surgery (T2). Stress levels remained high across all time points for one CT patient. Participants expressed only positive comments about the study; many found it useful to gauge how they were functioning through treatments. However, they reported that the pre-operative period was stressful and time pressured, and could understand why many women may not want to participate.

### Cytokines results

**Fig. 1:**
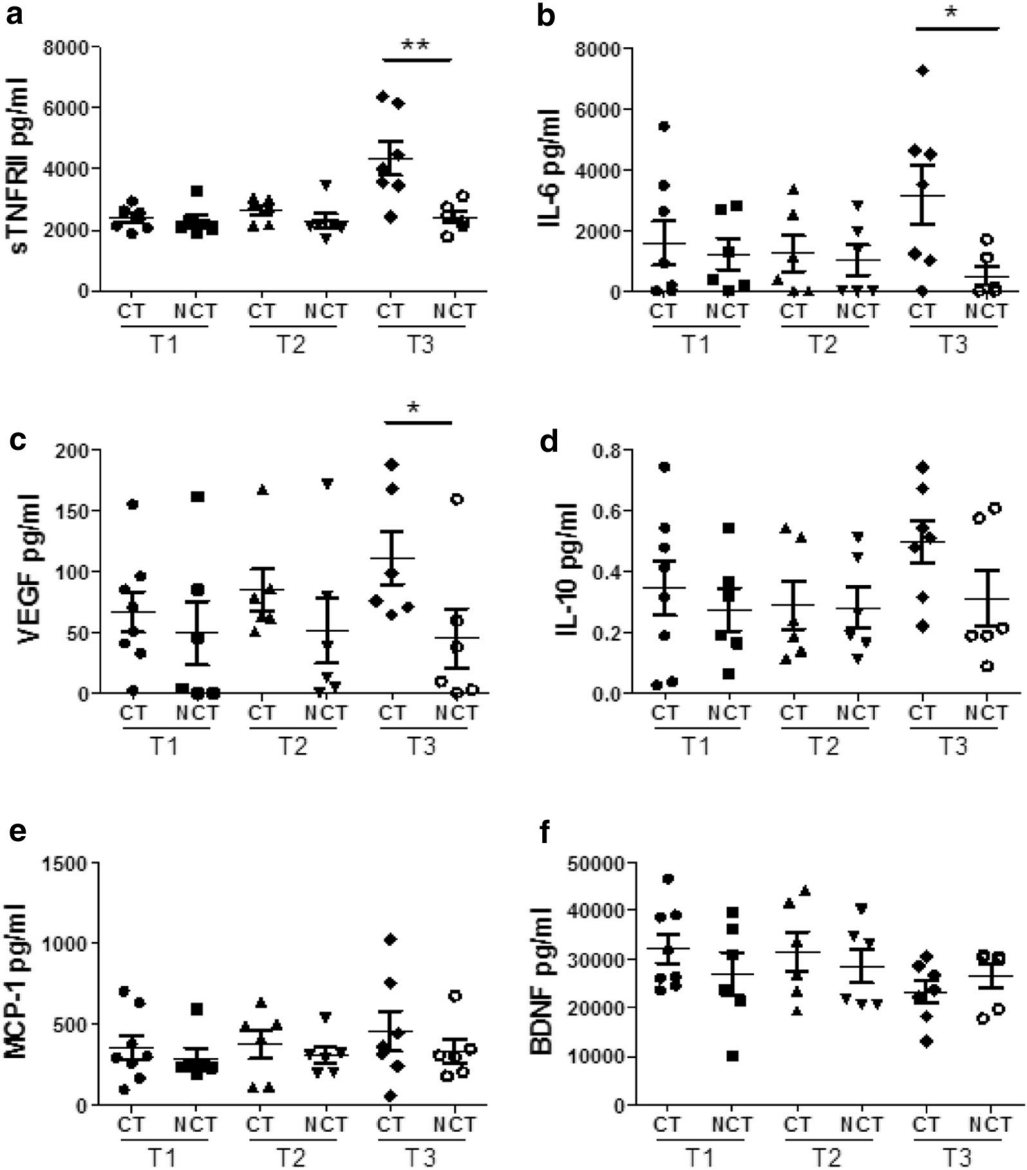
Levels of circulating cytokines are altered by treatment with chemotherapy. Serum was collected from breast cancer patients being treated with chemotherapy (CT) and those not undergoing chemotherapy (NCT). Serum levels of **a** sTNFRII, **b** IL-6, **c** VEGF, **d** IL-10, **e** MCP-1 and **f** BDNF were measured. Data for eight CT patients and six NCT patients is shown as the mean ± SEM (*p < 0.05; **p < 0.01)

All cytokine levels in NCT patients were unchanged across time and no changes were observed in CT patients between T1 and T2 (Fig. 1). In CT patients, significant elevations of sTNFRII (p = 0.0041), IL-6 (p = 0.0298) and VEGF (p = 0.013) were observed at T3 relative to NCT (Fig. 1a–c). An increase in IL-10 was also detected but this did not reach significance (Fig. 1d), whereas no changes were observed between the patient groups at any of the time points for MCP-1 and BDNF (Fig. 1e, f). Correlations with patient-reported fatigue and perceived cognitive function (PCI, PCA) were not significant.

### Neuroimaging results

**Fig. 2:**
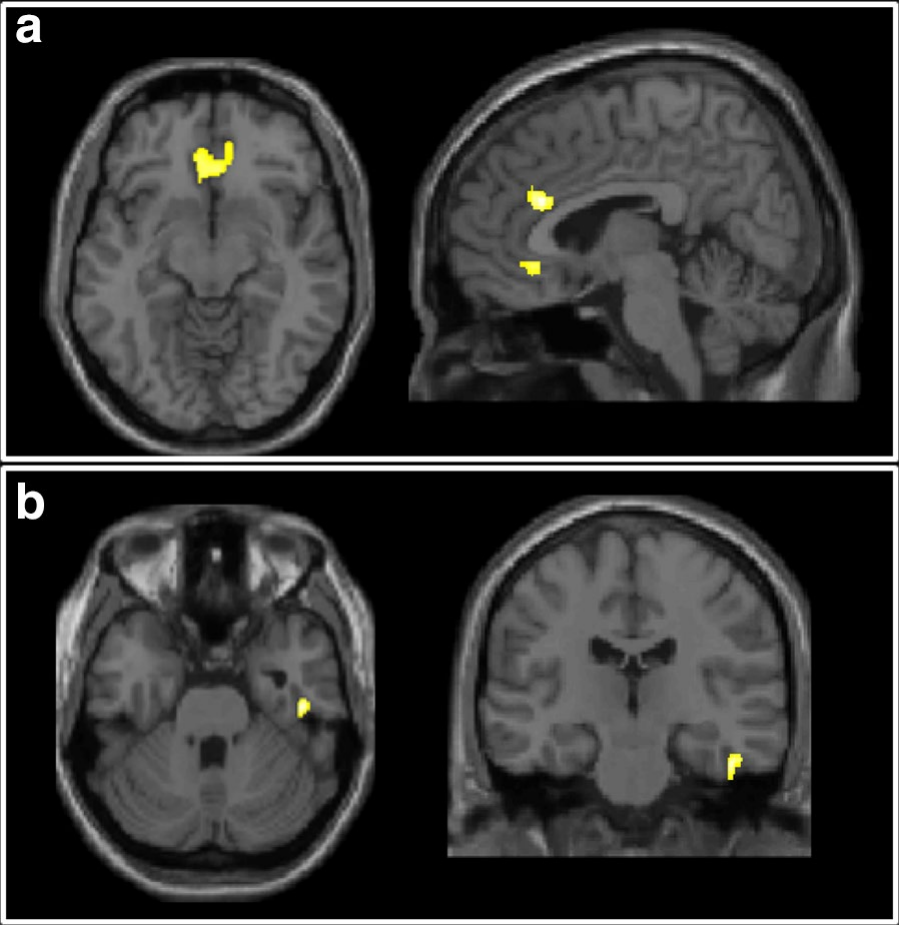
Grey matter volume results: two-way ANOVA. The areas of significant group-by-time interaction are *highlighted*, and overlaid onto a T1-weighted template image. **a** 2 clusters in the cingulate cortex; **b** the inferior temporal gyrus cluster

**Fig. 3:**
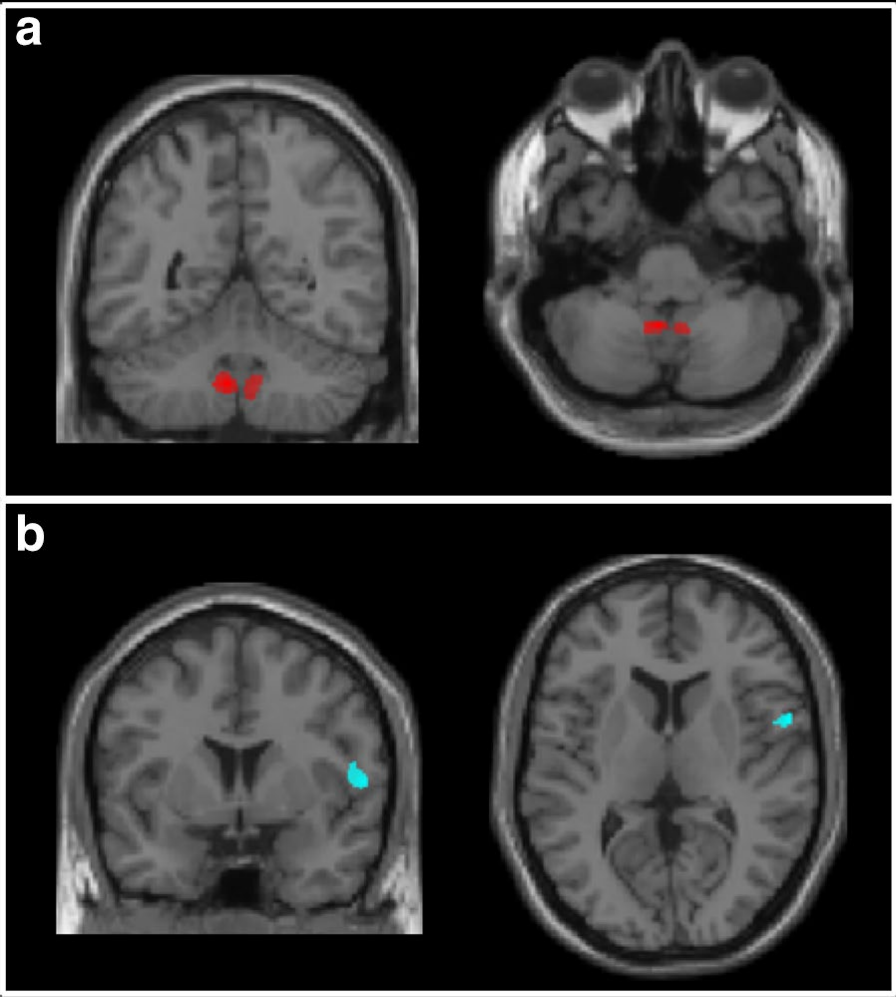
Grey matter volume results: correlations with cytokines. The *solid coloured areas* indicate the localisation of the significant correlation between grey matter volume and IL-6 (**a**) or sTNFRII (**b**). The clusters are overlaid onto a T1-weighted template image in standard space

Table 3 summarizes the location of grey matter clusters where a significant group-by-session interaction was found with respect to GMV. VBM analysis showed a significant group-by-session interaction, with CT patients showing larger volumetric reductions in the sub-genual and anterior midcingulate cortex (aMCC) as well as the inferior temporal gyrus (ITG) compared to NCT (Fig. 2; Table 3). Significant correlations were also found at T3 between GMV of the posterior insula and sTNFRII, and between cerebellar lobule IX volume (bilaterally) and IL-6 (Fig. 3).

**Table 3:** Grey matter volume results of the two-way ANOVA indicating the brain area, the spatial coordinates and the *p* value of the significant effect

Brain region	Montreal Neurological Institute coordinates			Peak T value	*p* value
	x	y	z		
Right inferior temporal gyrus	47	−20	−29	4.74	<0.001
Subgenual cingulate cortex	0	32	−6	4.38	<0.001
Right anterior midcingulate gyrus	8	29	22	4.53	<0.001

## Discussion

Pre-operative recruitment to a non-clinical breast cancer trial is challenging, and lengthy study procedures are not feasible for studies with limited recruitment timelines. A key factor in recruitment was timing. Time constraints and research burden were the main reasons for nonparticipation in our feasibility study. A reluctance to participate in research between breast cancer diagnosis and surgery has been reported elsewhere (Hepworth et al. 2011; Ashley et al. 2012). Another reason for declining the study was anxiety about the scheduled breast surgery. This is not uncommon and both diagnostic (e.g. excisional biopsy) and curative breast cancer surgery (e.g. mastectomy) are emotionally taxing producing heightened distress (Montgomery et al. 2003; Poole 1997). For the majority of women in our study stress levels reduced significantly following surgery. Interestingly, our cytokine analyses showed no significant change before and after surgery further supporting the case for approaching breast cancer patients for these types of studies in the post-operative period.

Our secondary objectives were met in part; there were no significant differences in QoL, levels of fatigue and perceived memory problems for women from pre to post-surgery. However at 6 months those who received chemotherapy reported more memory problems, greater fatigue and poorer QoL. Additionally, self-reported problems with memory and attention were associated with reported levels of fatigue, which may give reassurance to some patients that there was an explanation for their symptoms. The primary purpose of the study was to establish the feasibility of pre-operative assessment, so it was not powered to examine differences between groups for tasks of memory and attention, but individual change analysis showed most women performed well.

There were no significant changes in cytokines pre and post-surgery. Significant increases were observed in the serum levels of sTNFRII, IL-6 and VEGF following chemotherapy. These findings were supportive of prior studies which hypothesised that chemotherapy induced fatigue may be mediated by changes in inflammatory cytokines (Ahles and Saykin 2007). We did not observe any significant associations between increased cytokine levels and fatigue or subjective cognitive complaints most probably due to the small sample size; however such observations have been made by other researchers, (Bower et al. 2011; Ganz et al. 2013). In one cohort study, exposure to chemotherapy (i.e. ≤3 months after last cycle) was linked to elevations in sTNFRII, with the strongest associations between sTNFRII and fatigue in the chemotherapy exposed patients (Bower et al. 2011). Ganz et al. (2013) confirmed at follow-up in the same sample that higher baseline sTNFRII levels were significantly correlated to increased self-reported memory complaints but not to objective neuropsychological test results. These results support our observation of significant sTNFRII elevation in chemotherapy exposed patient sera. The near uniform increase in sTNFRII elevation in CT but not NCT patients suggests that sTNFRII is a useful marker of peripheral cytokine alterations following chemotherapy.

A recent study also confirmed the association between pro inflammatory cytokines (higher concentrations of IL-6 and IL-1ß) and more severe self-perceived cognitive disturbances (Cheung et al. 2013). Other research suggests further that cytokines and inflammatory markers can give rise to a cluster of cancer-related symptoms including fatigue, depression and stress, which are associated with cognitive changes (e.g. Bower et al. 2011; Lee et al. 2004; Meyers 2008). A comprehensive review noted that these symptoms are rarely controlled and concluded that the intermediary role of cytokines in post-chemotherapy cognitive impairment remains controversial (Ganz et al. 2013).

Research has shown neuroanatomical differences between breast cancer patients (pre-chemotherapy) and matched non-cancer controls, stressing the importance of the comparison group (Scherling et al. 2012). In our study, a larger reduction in the volume of the sub-genual and dorsal anterior cingulate as well as the inferior temporal gyrus (ITG) was observed for the chemotherapy group compared to the non-chemotherapy group. Sub-genual and dorsal aspects of the anterior midcingulate cortex (aMCC) have previously been reported to show functional alterations following experimentally-induced inflammation (Harrison et al. 2009a, b). Interestingly, changes in subgenual cingulate function and volume have been repeatedly demonstrated in idiopathic depression (Mayberg et al. 2005; Drevets et al. 1997). Furthermore, reductions in sub-genual cingulate reactivity to emotional stimuli also appears to mediate inflammation-induced mood change (Harrison et al. 2009a). Together, these findings suggest that post-chemotherapy cognitive changes may relate to subtle effects on mood and/or motivation and support our hypothesis that our observed grey matter changes may relate to chemotherapy-induced inflammation. The significance of our observed changes in ITG are less clear. The ITG’s primary function relates to visual object recognition and memory, however, a recent paper has reported greater atrophy of the right ITG in fatigued versus non-fatigued patients with multiple sclerosis (Rocca et al. 2014).

Relationships between circulating cytokine levels and regional brain volumes have been reported in breast cancer survivors. In one study of 20 breast cancer patients and 23 healthy controls, left hippocampal volumes were reduced significantly and IL-6 and TNF-α concentrations elevated in the breast cancer group and lower left hippocampal volume was associated with higher circulating TNF-α and lower IL-6 (Kesler et al. 2013). In our study, circulating sTNFRII inversely correlated with GMV in the right posterior insula (across both groups). Insula is believed to provide a cortical representation of bodily physiological state including inflammatory status (Critchley and Harrison 2013) and activity changes have been repeatedly shown to correlate with inflammation-induced fatigue (Harrison et al. 2009b, 2015). Finally, we also observed a significant correlation between cerebellar lobule IX volume (bilaterally) and IL-6. Little is currently known about the functional role of this lobule, but it has been suggested to form part of the default mode network (Habas et al. 2009), a network central to off-task ‘resting’ brain activity. However, given the small sample size of the current study, the significance of these observed correlations needs to be considered with caution and warrants further investigation.

In summary, the low acceptance rate showed that it is not feasible to recruit women for cognitive testing and brain imaging prior to breast cancer surgery. As there were few differences in patient reported outcomes, cognitive tests or changes in blood cytokine levels before and after surgery, we can be cautiously confident that the period post-surgery but pre-chemotherapy is a pragmatic and valid baseline assessment time point. The hypothesis that chemotherapy induced fatigue is mediated by a change in peripheral cytokine levels is supported by our pilot data and observed changes on brain scans, which may explain the symptoms often experienced by patients undergoing chemotherapy. A larger longitudinal study is now warranted to confirm these preliminary hypotheses and investigate whether some women are more susceptible than others to fatigue. Future studies may consider pharmaceutical and non-pharmacological interventions to combat fatigue to help ameliorate the symptoms of ‘chemo brain’.

## Figures and Tables

**Fig. 4:**
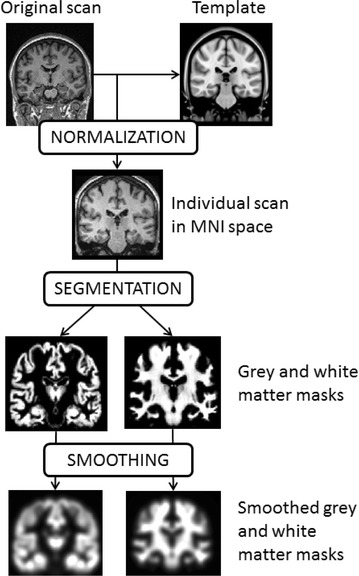
Flow diagram of the pre-processing steps in standard voxel-based morphometry

## Appendix 1

See Table 4.

**Table 4:** Overview of neuropsychological tests

Test	Ability measured	Outcome measure
National Adult Reading Test^a^ (Nelson and Wilson 1991; Crawford et al. 1989)	Premorbid intelligence	Total correct responses
Rey Auditory Verbal Learning Test^b^ (Schmidt 1996)	Verbal learning, recall, and retention	Supraspan (trial 1), total correct trials 1-5, delayed recall (trial 7)
Grooved Pegboard (Lezak 2012; Lafayette 1998)	Manual fine motor speed	Completion time, total number pegs placed with dominant and nondominant hand
Letter–number sequencing (Wechsler 1997)	Working memory capacity	Total correct responses
Double letter cancellation task (Lezak 2012)	Processing speed, attention	Completion time, total number of correct responses
Stroop Colour and Word Test (Golden 1978)	Cognitive flexibility, executive function	Total correct for word-reading, colour naming, and colour-word reading task (45 s for each tasks)
Controlled Oral Word Association Test^c^ (Benton et al. 1994; Shao et al. 2014)	Language, executive function	Total number of correct words produced within the given letter triad (1 min for each letter)

^a^Baseline (T1) only

^b^Alternate versions were used at each time point

^c^Two parallel sets of letters were used (C–F–L at T1 and T3, P–R–W at T2)

## Appendix 2

See Fig. 4.
